# Supplementary material for: Voltage control of magnetism in Fe3-xGeTe2/In2Se3 van der Waals ferromagnetic/ferroelectric heterostructures
Source: Nat Commun. 2023 Sep 12;14:5605. doi: 10.1038/s41467-023-41382-8 (PMC10497543; doi:10.1038/s41467-023-41382-8)
Supplement: Supplementary file 1 — Supplementary Information [file 41467_2023_41382_MOESM1_ESM.pdf]

Supplementary Information

Voltage control of magnetism in $Fe_{3-x}GeTe_2/In_2Se_3$ van der Waals ferromagnetic/ferroelectric heterostructures

Jaeun Eom[1,2], Inhak Lee[1], Jung Yun Kee[1,3], Minhyun Cho[4], Jeongdae Seo[5], Hoyoung Suh[6], Hyung-Jin Choi[7], Yumin Sim[8], Shuzhang Chen[9,10], Hye Jung Chang[6], Seung-Hyub Baek[7], Cedomir Petrovic[9,10], Hyejin Ryu[1], Chaun Jang[1], Young Duck Kim[4], Chan-Ho Yang[5], Maeng-Je Seong[8], Jin Hong Lee[1,*], Se Young Park[3,11,*], Jun Woo Choi[1,*]

[1] Center for Spintronics, Korea Institute of Science and Technology (KIST), Seoul 02792, Korea

[2] Department of Physics and Astronomy, Seoul National University, Seoul 08826, Korea

[3] Department of Physics, Soongsil University, Seoul 06978, Korea

[4] Department of Physics and Department of Information Display, Kyung Hee University, Seoul 02447, Korea

[5] Department of Physics, KAIST, Daejeon 34141, Korea

[6] Advanced Analysis Center, Korea Institute of Science and Technology (KIST), Seoul 02792, Republic of Korea

[7] Electronic Materials Research Center, Korea Institute of Science and Technology (KIST), Seoul 02792, Korea

[8] Department of Physics, Chung-Ang University, Seoul 06974, Korea

[9] Condensed Matter Physics and Materials Science Department, Brookhaven National Laboratory, Upton, New York 11973, USA

[10] Department of Physics and Astronomy, Stony Brook University, Stony Brook, New York 11794-3800, USA

[11] Origin of Matter and Evolution of Galaxies (OMEG) Institute, Soongsil University, Seoul 06978, Korea

Corresponding authors: Jin Hong Lee, Se Young Park, Jun Woo Choi

E-mail to Corresponding authors: jinhong.lee87@gmail.com, sp2829@ssu.ac.kr, junwoo@kist.re.kr

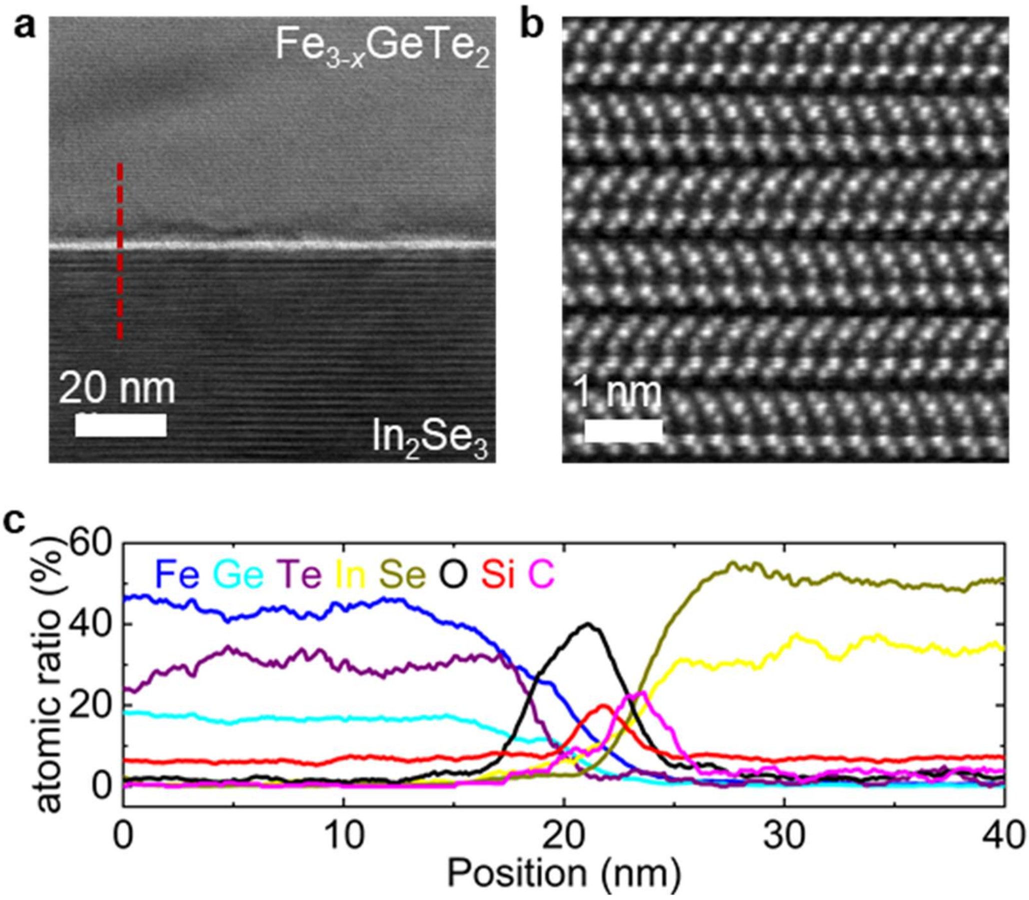

Fig. S1. The cross-sectional TEM images and EDS graphs of a FGT/IS heterostructure device. a) a cross-sectional TEM bright field image showing the interface between FGT and IS. The scale bar indicates 20 nm. b) a close-up atomically-resolved cross-sectional STEM HAADF image of the IS in the heterostructure. The scale bar indicates 1 nm. c) a graph of atomic ratio versus position from EDS data. The red dotted line in a) represents the positions in the horizontal axis of the graph.

The structure of the FGT/IS heterostructure device is characterized with cross-sectional TEM. The FGT and IS show layered structures with vdW gaps (Fig. S1a). The IS layer shows atomic alignments typical of a downward polarized monocrystalline 2H α-IS (Fig. S1b)[1,2]. We

also see the existence of a thin (~2 nm) and uniform layer consisting of lighter elements at the interface between FGT and IS (Fig. S1a). This interfacial layer is repeatedly and consistently observed in other similar FGT/IS heterostructure specimens that we fabricated and measured. We use EDS to identify and quantify elements across the interface (Fig. S1c). The interfacial layer has detectable quantities of carbon, silicon, and oxygen, all of which are also the full composition of PDMS. It may look as if the interfacial layer is mixed with FGT and IS according to the EDS graph (Fig. S1c), but judging from the clear boundaries of each layer (Fig. S1a), we believe it results from the relatively low resolution of the EDS measurement. Therefore, we suspect it is the residue from the vdW material transfer process using PDMS. We speculate that this interfacial layer might act as an adhesive mediator of strain across the interface. The consistent voltage-induced Raman peak redshifts (in-plane tensile strain) in both the FGT and IS (see main text Fig. 3) confirm this.

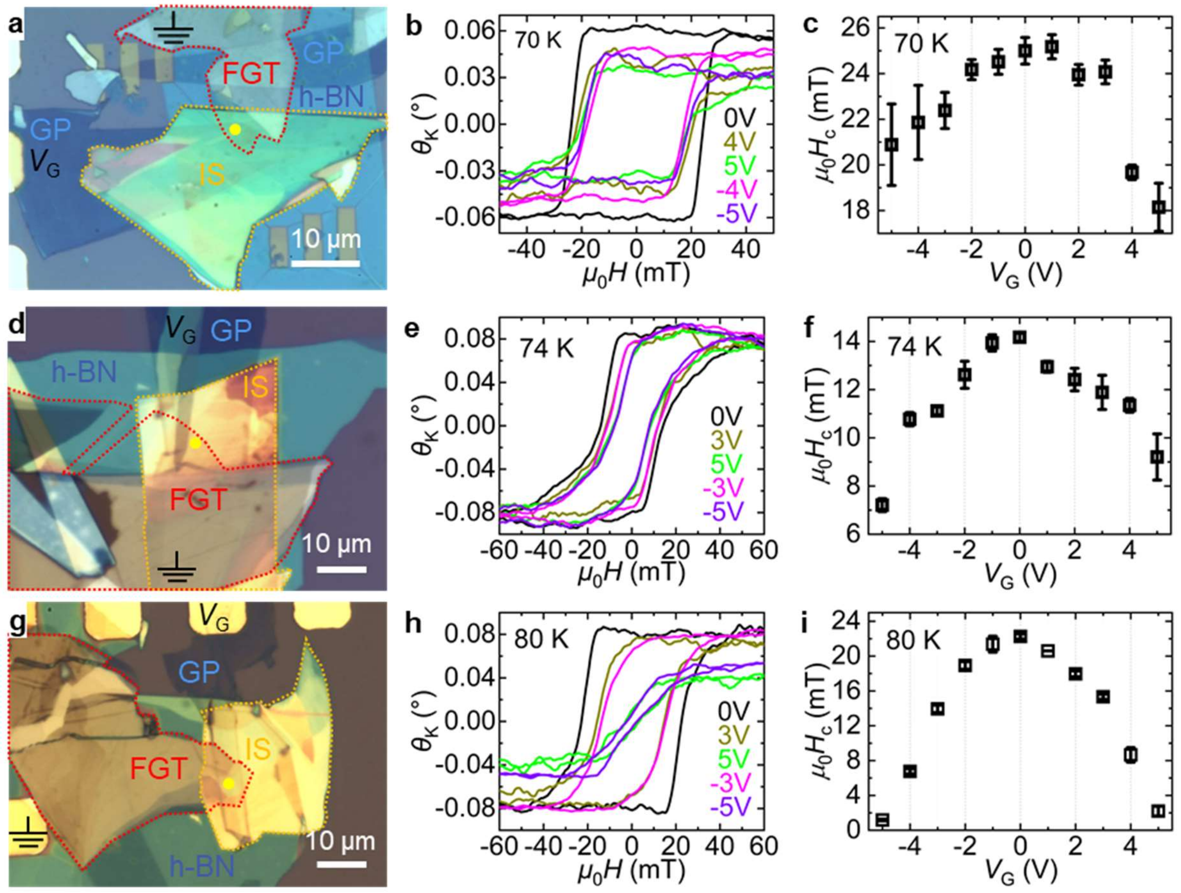

Fig. S2. V_G-dependent H_c modulation in other FGT/IS devices.

a) Optical microscopy (OM) image of the FGT(12 nm)/IS(100 nm) heterostructure device. b) V_G-dependent *M*-*H* loops and c) H_c plotted as a function of V_G for the device shown in (a). Measurements at 70 K.

d) OM image of the FGT(10 nm)/IS(40 nm) device. e) V_G-dependent *M*-*H* loops and f) H_c plotted as a function of V_G for the device shown in (d). Measurements at 74 K.

g) OM image of the FGT(20 nm)/IS(50 nm) device. h) V_G-dependent *M*-*H* loops and i) H_c plotted as a function of V_G for the device shown in (g). Measurements at 80 K.

The plot legends in b), e), and f) are in sequential order. The error bars represent the standard deviation of multiple measurement data.

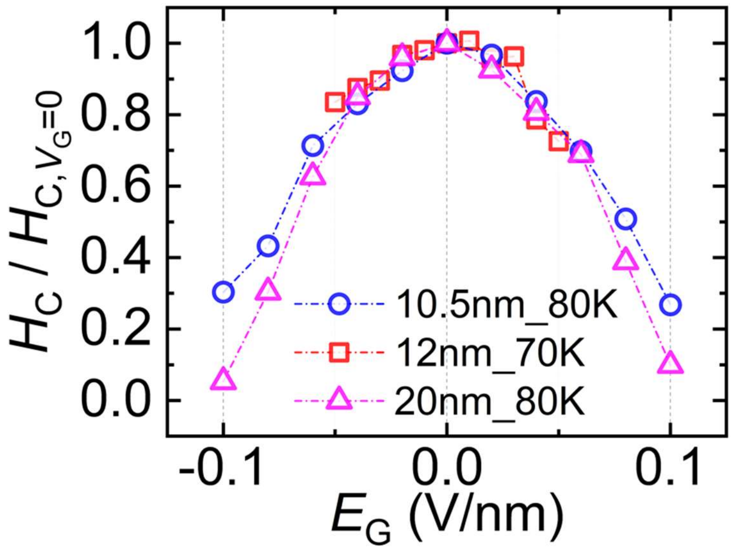

Fig. S3. FGT-thickness-dependent H_c modulation. H_c plotted as a function of E_G ($\equiv V_G$ / IS-thickness) for the FGT/IS devices shown in Fig. 1b, Fig. S2a, Fig. S2g. The H_c values are normalized to the H_c at E_G = 0 V for each heterostructure device. The labels indicate the FGT thickness and measurement temperature.

The voltage effect does not show any significant FGT-thickness-dependence in the thickness range 10.5~20 nm. This implies that the voltage-induced-piezo-strain transfer between the IS and FGT is effective up to ~20-nm-thick FGT layers. Note that in all the heterostructure devices studied, FGT thickness (10.5~20 nm) << IS thickness (50~100 nm). In the case the polarization-induced IS surface charge is the origin of the V_G-induced H_c modulation, the effect would show a more obvious FGT-thickness-dependence.

For FGT thickness > 30 nm, the magnetic stripe domain phase appear[3,4], with characteristic spilt *M*-*H* loops which makes it difficult to define H_c, and hence, only thin (< 20 nm) FGT layers are used for this study.

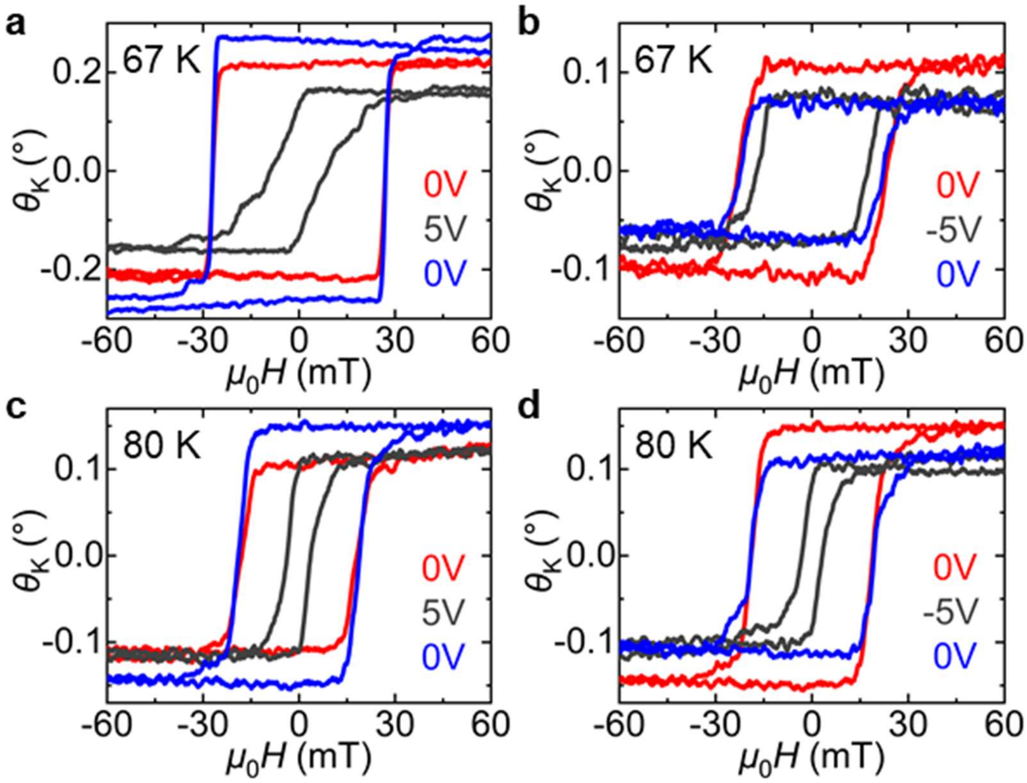

Fig. S4. Non-remanent voltage effect on MOKE hysteresis loops with V_G off, on, and then off for a) 67 K, positive V_G; b) 67 K, negative V_G; c) 80 K, positive V_G; and d) 80 K, negative V_G. The order of the legend in each plot is chronological. The H_c decreases during V_G application, but returns to its original value at zero bias when the V_G is tuned off, clearly showing the reversibility and non-remanence of the V_G–induced H_c modulation. The small change in the MOKE signal (Kerr rotation) before and after voltage application is likely due to sample drifts during the measurements. The MOKE laser beam size is comparable to the heterostructure device size (see Methods in main text). The plot legends are in sequential order.

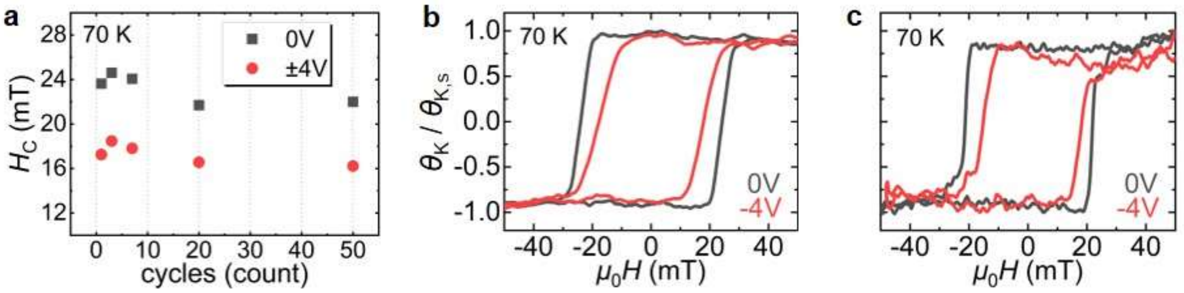

Fig. S5. Reversibility test of V_G–dependent H_c change in FGT/IS device measured over 50 cycles. a) The non-remanent and reversible V_G–induced H_c change is consistently observed after 50 cycles of voltage application. The cycle measurements are performed on the FGT/IS device shown in Fig. S2a-c. Each cycle consists of voltage applications up to V_G = ± 4 -5 V, and back to V_G = 0 V. b-c) The magnetic hysteresis loops with V_G = 0 V and V_G = -4 V for b) the 1st cycle and c) the 50th cycle further demonstrate the stability of the device.

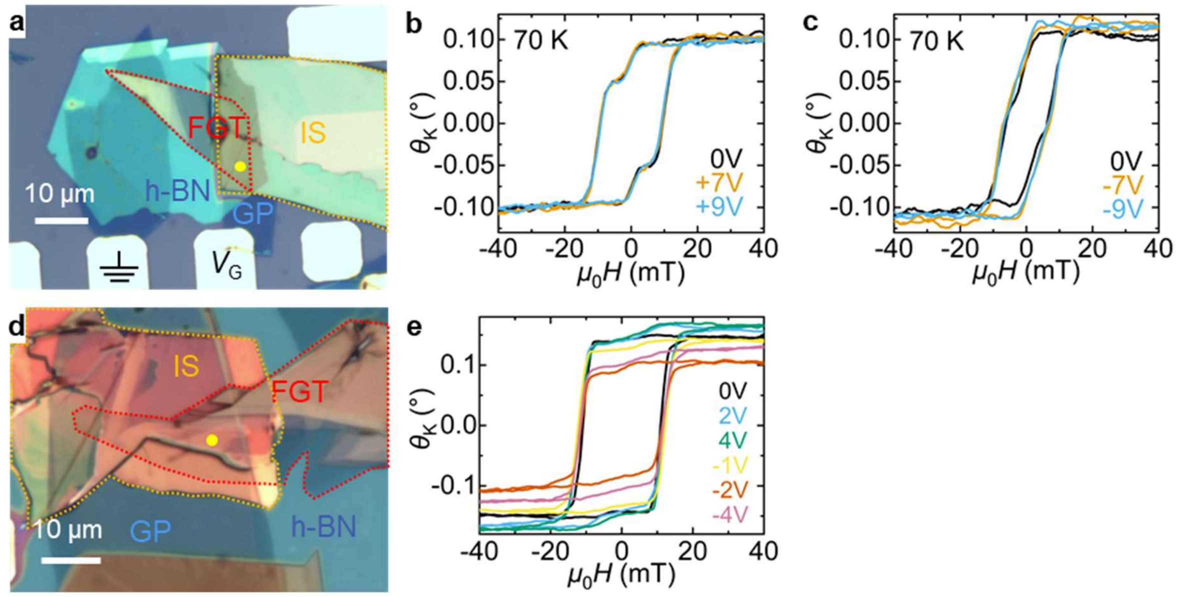

Fig. S6. Voltage-dependent magnetic properties of a FGT/β-In_2Se_3 heterostructure device. In contrast to the ferroelectric α-In_2Se_3, β-In_2Se_3 is *non-ferroelectric*. a) OM image of a FGT(14 nm)/β-In_2Se_3(300 nm) heterostructure device. b-c) The V_G-dependent magnetic hysteresis loops of the device shown in a) for b) positive V_G, and c) negative V_G. The FGT flake in this device has many stepped regions and b) and c) are measured on different regions within the FGT flake. Note that the β-In_2Se_3 here is thicker than the typical α-In_2Se_3 we used for our experiments, and hence, we applied voltages up to $V_G = \pm 9$ V. d) OM image of a FGT(12 nm)/β-In_2Se_3(60 nm) heterostructure device. e) The V_G-dependent magnetic hysteresis loops of the device shown in d). Measurements done at 70 K. The plot legends are in sequential order.

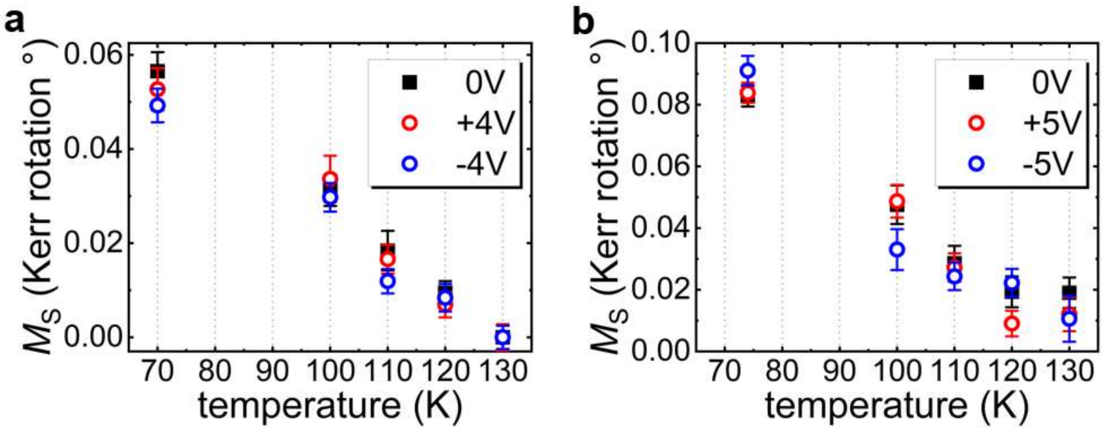

Fig. S7. V_G–dependent M-T plots for FGT/IS heterostructure devices. The saturation MOKE signal (Kerr rotation angle) is plotted as a function of temperatures for V_G = 0 V and V_G = ± 4 or 5 V. a) M-T plot at V_G = 0 V and V_G = ± 4 V in the device shown in Fig. S2a. b) M-T plot at V_G = 0 V and V_G = ± 5 V in the device shown in Fig. S2d. The Curie temperature (T_C) of the FGT flakes can be estimated from the temperature at which the MOKE signal (sensitivity ~0.01°) vanishes. The T_C of the FGT flakes are ≈ 120 K at V_G = 0 V, and the T_C shows little change with voltage application (V_G = ±4 or 5 V) for both FGT/IS devices. The error bars represent the standard deviation of multiple measurement data.

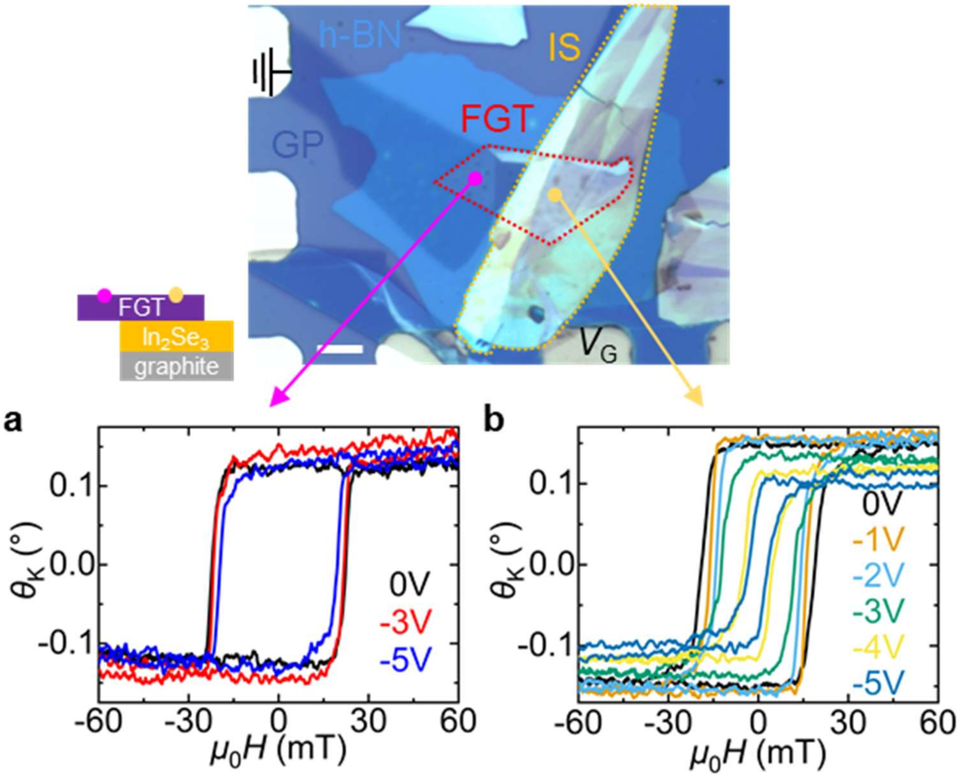

Fig. S8. Comparison of V_G–dependent hysteresis loops measured on a) FGT/graphite and b) FGT/IS/graphite. MOKE measurements are performed on different regions within the same metallic FGT flake, such that the same level of electric current is flowing in both measurements. a) V_G–dependent hysteresis loops of FGT that is *not* on top of IS (pink spot in the top OM image), which show little V_G effect. b) V_G–dependent hysteresis loops measured on FGT that overlaps the IS (yellow spot in OM image), which show significant V_G effect (identical plot with Fig. S2b). The plot legends are in sequential order.

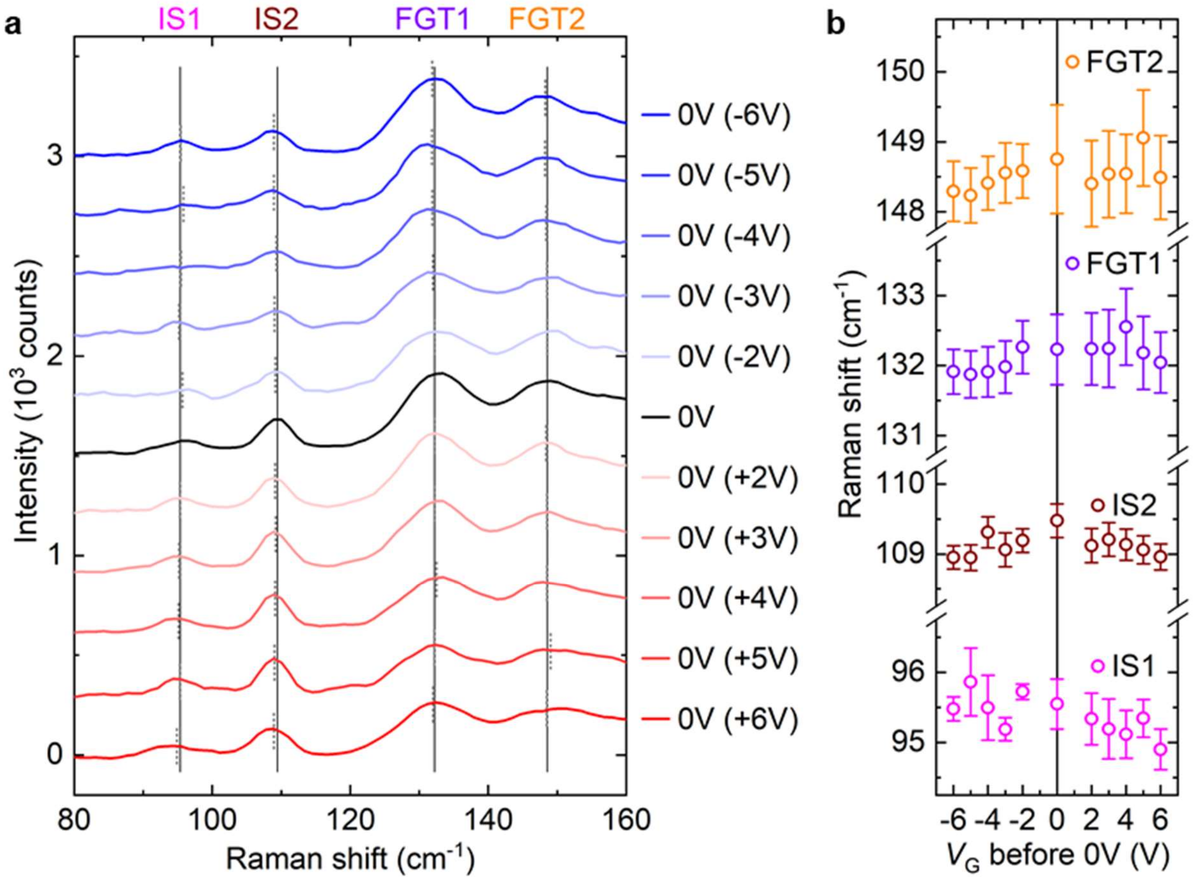

Fig. S9. Raman shifts at zero bias after voltage application. a) A series of zero-bias Raman spectra of the FGT/IS heterostructure after applying the V_G in parenthesis, measured at 70 K. The four fitted Raman peak positions at zero bias are shown as solid black lines and the peak positions for each voltage application is shown as gray dotted lines. b) A summary of the zero-bias Raman peak positions shown in a), with the horizontal axis indicating the applied V_G before measuring the Raman shifts at zero-bias. The error bars are defined in the main text Methods. Unlike the V_G-induced redshift seen in main text Fig. 3, zero-bias Raman peak positions have little dependency on the V_G applied prior to the zero-bias measurements. This implies the absence of strain at zero bias, i.e., the voltage effect is non-remanent, supporting the non-remanent H_c change observed in Fig. S3.

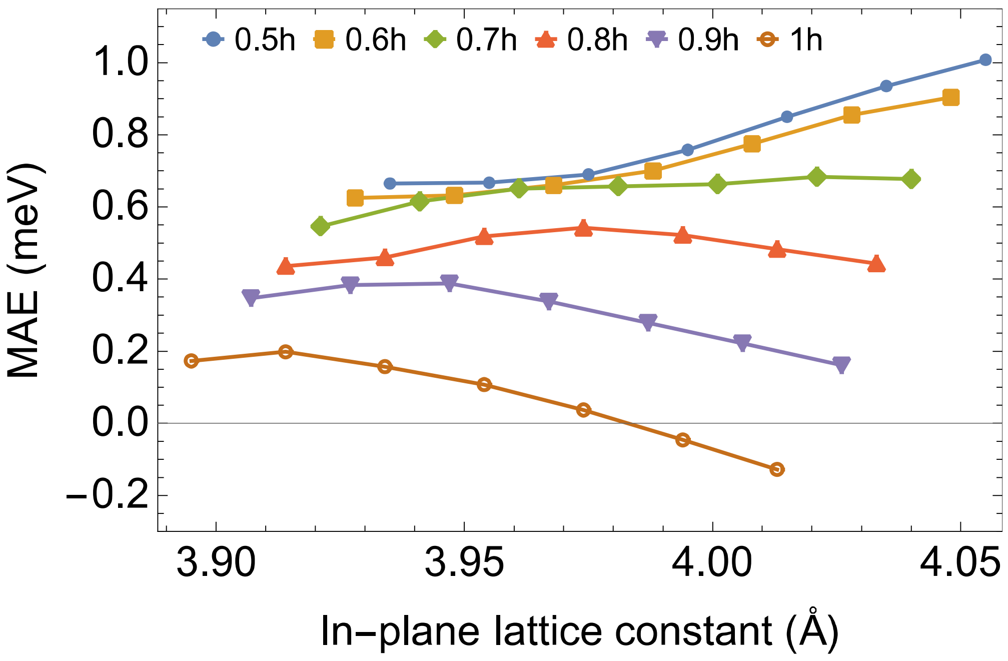

Fig. S10. MAE/Fe (in meV) as a function of in-plane lattice constant for various doping concentrations. As explained in the main text, the FGT sample we used in the experiments correspond to the 1h/f.u. case. The in-plane lattice constants in the plot are chosen corresponding to the in-plane strain between -1.5% and 1.5% relative to the experimental lattice constant, which is the fourth data-point for each doping concentration plot. There is overall decrease in MAE as hole doping increases. We find that the dependence of MAE with respect to in-plane strain changes with hole doping. For doping smaller than 0.8h/f.u. MAE increases (decreases) with tensile (compressive) strain whereas for the doping larger than 0.8h/f.u. MAE shows opposite behaviour. For 0.8h/f.u. MAE decreases both for tensile and compressive strain. We note that for doping less than 1h/f.u. MAE values maintain positive. However, for 1h/f.u. doping, the decrease in MAE is about 65% reduction in MAE for 0.5% tensile strain (a = 3.97 Å) and even induces sign change in MAE from out-of-plane to in-plane anisotropy for 1% tensile strain (a = 3.99 Å).

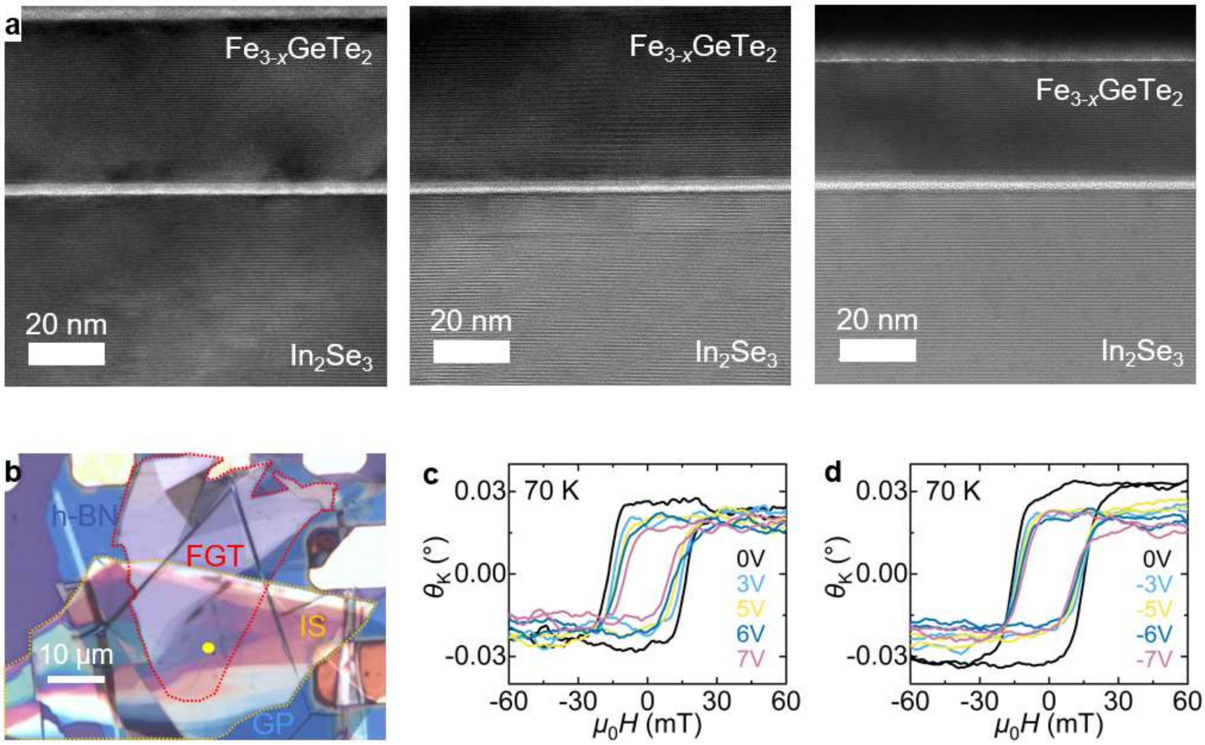

Fig. S11. Structural and magnetic properties of FGT/IS heterostructure devices fabricated by dry transfer using gel-pak. a) The cross-sectional TEM images of FGT/IS heterostructure devices fabricated by dry-transfer using gel-pak. We see the consistent presence of residual polymer layer at the FGT-IS interface. b) Optical microscopy (OM) image of the FGT/IS device fabricated using gel-pak. c-d) V_G-dependent *M-H* loops for c) positive V_G, and d) negative V_G. V_G-dependent H_c decrease is observed. The plot legends are in sequential order.

References

1. Lv, B. et al. Layer-dependent ferroelectricity in 2H-stacked few-layer α-In_2Se_3. *Mater. Horiz.* **8,** 1472-1480 (2021).
2. Cui, C. et al. Intercorrelated in-plane and out-of-plane ferroelectricity in ultrathin two-dimensional layered semiconductor In_2Se_3. *Nano Lett.* **18,** 1253-1258 (2018).
3. Li, Q. et al. Patterning-Induced Ferromagnetism of Fe3GeTe2 van der Waals Materials beyond Room Temperature. *Nano Lett*. **18**, 5974-5980 (2018).
4. Park, S. Y. et al. Controlling the magnetic anisotropy of the van der Waals ferromagnet Fe_3GeTe_2 through hole doping. *Nano Lett*. **20**, 95-100 (2020).
